# Editorial: Immunotherapeutic strategies to target cancer stem cells: state of the art in basic research to clinical application

**DOI:** 10.3389/fimmu.2024.1490569

**Published:** 2024-09-23

**Authors:** Amirhesam Babajani, Marzieh Naseri, Faezeh Vakhshiteh, Roya Ghods, Zahra Madjd

**Affiliations:** ^1^ Oncopathology Research Center, Iran University of Medical Sciences (IUMS), Tehran, Iran; ^2^ Department of Pharmacology, School of Medicine, Shahid Beheshti University of Medical Sciences, Tehran, Iran; ^3^ Department of Developmental, Molecular and Chemical Biology, Tufts University School of Medicine, Boston, MA, United States; ^4^ Department of Molecular Medicine, Faculty of Advanced Technologies in Medicine, Iran University of Medical Sciences (IUMS), Tehran, Iran

**Keywords:** cancer stem cells, immunotherapy, tumor microenvironment, CSC plasticity, immune evasion, biomarkers, extracellular vesicles (EVs), vaccine

Cancer remains one of the most daunting challenges in modern medicine, and while advances in treatment have brought hope to many, the battle is far from over. A pivotal concept in oncology is the existence of Cancer Stem Cells (CSCs), a subpopulation of tumor-forming cells known for their capacity for self-renewal, differentiation, and quiescence. They are responsible for many of the neoplastic and invasive characteristics of malignancies, such as immune escape, angiogenesis, multidrug resistance, and recurrence (1). The cancer stem cell plasticity concept—where CSCs can dynamically switch between CSC and non-CSC states—further complicates targeting these cells. This plasticity allows CSCs to evade treatments that might otherwise be effective, necessitating a more comprehensive approach that targets both CSCs and non-CSCs within tumors (2). Targeting CSCs is crucial in developing more effective cancer treatments, particularly in the burgeoning field of cancer immunotherapy.

Immunotherapy, which harnesses the immune system to fight cancer, has revolutionized the treatment landscape. Notably, the introduction of checkpoint inhibitors like ipilimumab and nivolumab for melanoma treatment has marked a new era in cancer therapy (3). However, despite these successes, targeting CSCs remains a challenge, as these cells are adept at evading the immune system and driving tumor recurrence and treatment resistance. As we delve into this issue, integrating CSC-targeted strategies into immunotherapy becomes increasingly apparent, with recent studies providing both challenges and insights that can guide future research.

One such study by Holmström et al. examined the therapeutic cancer vaccination against mutant calreticulin (CALR) in patients with CALR-mutant (CALRmut) myeloproliferative neoplasms (MPN). Some studies have reported that CALR is highly expressed in CSCs, which can be considered a poor survival prognostic factor and immune surveillance (4, 5). The study of Holmströms’s team found that the vaccine failed to demonstrate significant clinical activity despite inducing strong T-cell responses against the mutant CALR. The research revealed that CALRmut-specific T cells did not significantly increase in the bone marrow post-vaccination, suggesting a potential disconnect between the immune response generated and its effective deployment within the tumor microenvironment. This finding underscores a critical challenge in cancer immunotherapy: the need for immune cells not only to recognize and target CSCs but also to infiltrate and function within the tumor microenvironment effectively. This issue is particularly pertinent when considering CSCs, which reside in specialized niches within tumors and are often protected from immune attacks.

Thus, the interaction between CSCs and the tumor immune microenvironment is a crucial area of focus. As another study by Li et al. highlights, extracellular vesicles (EVs) and exosomes play a significant role in communicating CSCs and immune cells, influencing the tumor immune microenvironment and promoting conditions favorable for CSC survival and propagation. This interplay is a double-edged sword: while it allows CSCs to evade the immune system, it also presents an opportunity for therapeutic intervention. By disrupting or modulating the EV-mediated communication between CSCs and immune cells, it may be possible to render CSCs more vulnerable to immune attacks, enhancing the efficacy of immunotherapy.

Researches by Kavianpour et al. further underscore the potential of immunotherapy for acute myeloid leukemia (AML), a particularly aggressive form of cancer with a high risk of relapse. Cell-based immunotherapies, including chimeric antigen receptor (CAR) T-cell therapy, T-cell receptor (TCR)-engineered T-cell therapy, and natural killer (NK) cell-based therapies, have shown promise in improving outcomes for AML patients (6). These therapies enhance the immune system’s ability to target and eliminate cancer cells, including CSCs. However, the complexity and heterogeneity of AML pose significant challenges, particularly in identifying optimal target antigens and mitigating the risks of on-target/off-tumor toxicity. Despite these challenges, the progress made in this area is encouraging, offering hope that similar strategies could be applied to other cancers where CSCs play a pivotal role.

As the integration of Omics into CSCs targets therapeutic approaches, the study of Jafari et al. resonates with research into cervical cancer (CC), another area where the role of CSCs is becoming increasingly recognized. While screening programs have reduced mortality rates, treatment resistance and recurrence remain significant issues, often driven by CSCs. Recent proteomics studies have identified biomarkers that could aid in diagnosing and treating CC, but the challenge remains in targeting CSCs effectively. These cells contribute to therapy resistance and metastasis, making them a critical target for improving patient outcomes. The ability of CSCs to evade treatment and drive recurrence is a significant barrier to the success of both traditional therapies and immunotherapies. Please add all references.

To effectively target CSCs, the initial step involves identifying potential and potent biomarkers. These biomarkers may include surface markers, signaling pathways, ABC transporters, and pathways that interfere with CSCs. Additionally, targeting the CSC niche, which serves as a host for immunoreactions against tumors, presents a promising therapeutic strategy. Furthermore, innovative approaches such as anti-angiogenic therapies and differentiation-based treatments offer novel avenues for targeting CSCs. Using stem cells also can be a potential therapeutic option to overcome the challenge of the CSC population in tumors. Previous studies have indicated stem cells’ targeted anti-neoplastic and immunomodulatory potentials (7, 8). Besides, preconditioning healthy stem cells with other anti-cancer agents can increase their anti-neoplastic effects (9). In addition, exosomes derived from CSC-enriched spheroids and monoclonal antibodies against CSC antigens represent additional avenues for targeting CSCs (10). These strategies aim to disrupt the tumor microenvironment and prevent cancer recurrence by eliminating CSCs and their progeny.

In conclusion, while significant progress has been made in cancer immunotherapy, the challenge of effectively targeting CSCs remains a critical obstacle. The studies discussed here highlight both the potential and the pitfalls of current approaches, emphasizing the need for continued research into the mechanisms by which CSCs evade the immune system and drive tumor recurrence. By focusing on the interplay between CSCs and the tumor immune microenvironment and by developing more targeted and personalized therapies, we can move closer to the goal of not just treating cancer but curing it. The future of cancer treatment lies in integrating CSC-targeted strategies into immunotherapy, offering hope for more effective and durable therapies for patients worldwide.

## References

[1] Batlle E Clevers H. Cancer stem cells revisited. Nat Med. (2017) 23:1124–34. doi: 10.1038/nm.4409 28985214

[2] Bhat GR Sethi I Sadida HQ Rah B Mir R Algehainy N. Cancer cell plasticity: from cellular, molecular, and genetic mechanisms to tumor heterogeneity and drug resistance. Cancer Metastasis Rev. (2024) 43:197–228. doi: 10.1007/s10555-024-10172-z 38329598 PMC11016008

[3] Larkin J Chiarion-Sileni V Gonzalez R Grob J-J Rutkowski P Lao CD. Five-year survival with combined nivolumab and ipilimumab in advanced melanoma. N Engl J Med. (2019) 381(16):1535–46. doi: 10.1056/NEJMoa1910836 31562797

[4] Matsukuma S Yoshimura K Ueno T Oga A Inoue M Watanabe Y. Calreticulin is highly expressed in pancreatic cancer stem-like cells. Cancer Sci. (2016) 107:1599–609. doi: 10.1111/cas.13061 PMC5132278 27561105

[5] Fujiwara Y Tsunedomi R Yoshimura K Matsukuma S Fujiwara N Nishiyama M. Pancreatic cancer stem-like cells with high calreticulin expression associated with immune surveillance. Pancreas. (2021) 50:405–13. doi: 10.1097/MPA.0000000000001772 PMC8041567 33835973

[6] Koedam J Wermke M Ehninger A Cartellieri M Ehninger G. Chimeric antigen receptor T-cell therapy in acute myeloid leukemia. Curr Opin Hematol. (2022) 29:74–83. doi: 10.1097/MOH.0000000000000703 35013048 PMC8815830

[7] Bolouri M-R Ghods R Zarnani K Vafaei S Falak R Zarnani A-H. Human amniotic epithelial cells exert anti-cancer effects through secretion of immunomodulatory small extracellular vesicles (sEV). Cancer Cell Int. (2022) 22:1–18. doi: 10.1186/s12935-022-02755-z 36307848 PMC9616706

[8] Babajani A Soltani P Jamshidi E Farjoo MH Niknejad H. Recent advances on drug-loaded mesenchymal stem cells with anti-neoplastic agents for targeted treatment of cancer. Front Bioeng Biotechnol. (2020) 8:748. doi: 10.3389/fbioe.2020.00748 32793565 PMC7390947

[9] Babajani A Manzari-Tavakoli A Jamshidi E Tarasi R Niknejad H. Anti-cancer effects of human placenta-derived amniotic epithelial stem cells loaded with paclitaxel on cancer cells. Sci Rep. (2022) 12:1–17. doi: 10.1038/s41598-022-22562-w 36307463 PMC9616866

[10] Naseri M Zöller M Hadjati J Ghods R Ranaei Pirmardan E Kiani J. Dendritic cells loaded with exosomes derived from cancer stem cell-enriched spheroids as a potential immunotherapeutic option. J Cell Mol Med. (2021) 25:3312–26. doi: 10.1111/jcmm.16401 PMC8034455 33634564

